# Integrating Surrounding Vehicle Information for Vehicle Trajectory Representation and Abnormal Lane-Change Behavior Detection

**DOI:** 10.3390/s23249800

**Published:** 2023-12-13

**Authors:** Da Xu, Mengfei Liu, Xinpeng Yao, Nengchao Lyu

**Affiliations:** 1 Intelligent Transportation Systems Research Center, Wuhan University of Technology, Wuhan 430063, China; xuda104971220387@whut.edu.cn (D.X.); lnc@whut.edu.cn (N.L.); 2 Shandong Hi-Speed Group Innovation Research Institute, Jinan 250014, China; yaoxinpeng2005@163.com; 3 Engineering Research Center of Transportation Information and Safety, Ministry of Education, Wuhan 430063, China

**Keywords:** driving behavior, lane change, abnormal behavior detection, trajectory representation, LGBM algorithm

## Abstract

The detection of abnormal lane-changing behavior in road vehicles has applications in traffic management and law enforcement. The primary approach to achieving this detection involves utilizing sensor data to characterize vehicle trajectories, extract distinctive parameters, and establish a detection model. Abnormal lane-changing behaviors can lead to unsafe interactions with surrounding vehicles, thereby increasing traffic risks. Therefore, solely focusing on individual vehicle perspectives and neglecting the influence of surrounding vehicles in abnormal lane-changing behavior detection has limitations. To address this, this study proposes a framework for abnormal lane-changing behavior detection. Initially, the study introduces a novel approach for representing vehicle trajectories that integrates information from surrounding vehicles. This facilitates the extraction of feature parameters considering the interactions between vehicles and distinguishing between different phases of lane-changing. The Light Gradient Boosting Machine (LGBM) algorithm is then employed to construct an abnormal lane-changing behavior detection model. The results indicate that this framework exhibits high detection accuracy, with the integration of surrounding vehicle information making a significant contribution to the detection outcomes.

## 1. Introduction

Lane-changing (LC) is one of the fundamental and indispensable driving behaviors of vehicles. When drivers aim to achieve more favorable driving conditions or perform diverging or merging operations, lane-changing behavior occurs [1]. During a lane-changing maneuver, drivers must monitor the traffic conditions in adjacent lanes and make decisions based on relative speed and distance; therefore, the driver’s field of vision involves extensive scanning of visual hazards from the front, sides, and even rear. They need to sift through information and make quick judgments. If the driver’s perception and operation are accurate and appropriate during a lane-changing behavior, it will be completed safe. However, in reality, the driver’s cognitive load significantly increases during the lane-changing process, making driving operations more error-prone and leading to driving risks [2,3,4,5]. Studies have confirmed that unsafe lane-changing behavior is one of the main causes of traffic instabilities, such as decreases in road capacity and speed, thus drawing increasing attention to lane-changing research in recent years [6,7,8].

Human factors are the primary causes of unsafe driving behaviors. Abnormal driving behaviors resulting from psychological states and attitudes can easily lead to traffic risks and even accidents [9,10,11]. Therefore, detecting and identifying abnormal driving behaviors, including abnormal lane-changing behavior, and subsequently regulating drivers’ driving habits through supervision, training, and education can reduce the occurrence of hazardous behaviors, thereby lowering the risk of road travel [12].

In recent years, artificial intelligence (AI) technology has accelerated the development of Intelligent Transportation Systems (ITS), with Machine Learning (ML) and Deep Learning (DL) techniques being widely applied in the detection of abnormal driving behaviors [13,14]. According to statistics in the field of driving behavior detection, ML algorithms are the most extensively used, accounting for 62.66%, followed by DL algorithms at 32.91% and statistical methods at 4.43% [15]. The use of ML and DL techniques has been proven as effective for driving behavior detection, leading to a better understanding of the causes of aberrant driving and an enhancement of road safety.

Regarding data sources, research on driving behavior mainly focuses on two aspects: objective and subjective measurement. Subjective measurement is primarily in the form of questionnaires, with The Manchester Driver Behavior Questionnaire (DBQ) being the most representative [16,17]. However, questionnaire surveys may introduce human subjectivity, leading to biases. Objective measurement can obtain quantitative data, and it comes in three main forms: driving simulator studies (DSS), field driving studies (FDS), and naturalistic driving studies (NDS) [18,19,20]. DSS collects simulated driving data from drivers on simulators. NDS uses unobtrusive measurement methods to record detailed information about the driver, vehicle, and surrounding environment; commonly used sensors include GPS, accelerometers, smartphone sensors, and OBDII sensors, among others [21,22,23]. However, in these two testing methods, drivers are consciously engaged in experimental testing, leading to deviations from actual driving performance. FDS uses equipment such as cameras and radar to monitor driver behavior at fixed points, encoding and recording driving behavior data; in comparison, it can obtain more objective and larger-scale data [24,25]. With the continuous development of Vehicle-to-Infrastructure (V2I) technology, road perception units, represented by high-definition cameras, can promptly acquire motion trajectory information, such as the position and speed of road vehicles. Through advanced communication technology, these units provide real-time road information to travelers and road regulatory authorities. This not only serves as a data source for the study of driving behavior but also establishes the foundational conditions for the application of abnormal behavior detection in road supervision [26,27,28,29].

In summary, video data and ML algorithms are feasible for detecting abnormal lane-changing behavior. Therefore, this study utilizes road video data and employs the approach of ML algorithms to conduct research on abnormal lane-changing behavior detection, aiming to serve the management and law enforcement of road traffic. Generally, there are two core tasks when using ML algorithms for abnormal lane-changing behavior detection: trajectory representation and behavior detection. Among these, trajectory representation serves as the foundation, extracting feature metrics from the trajectory to serve as input for the detection model. In many studies, the vehicle trajectory is directly represented through a coordinate vector and its variants [30]. Johnson et al. pioneered the description of object motion using a flow vector (x, y, dx, dy) [31]. Building upon this, subsequent research incorporated more information into the trajectory, such as speed, angle, position, etc., forming higher-dimensional coordinate vector representations [32,33]. Trajectory representation based on curve fitting has also been applied; common practice involves using low-order curves to approximate trajectories, such as using cubic B-spline curves for approximation [34]. However, methods based on curve fitting have obvious limitations, as the information extracted from the approximated trajectory shape is quite limited, making it difficult to discern underlying behavioral patterns. In recent years, deep learning methods have gradually gained widespread application in vehicle trajectory representation [35]. Lv et al. transformed trajectories into images and used convolutional neural networks to extract features from them [36]. Tang et al. employed hidden Markov models to learn and train trajectories in taxi route prediction [37]. Liang et al. used an Long Short-Term Memory (LSTM) encoder–decoder model to represent each trajectory as a series of intersections and related movement directions, which were then fed into the network for future trajectory generation [38]. However, despite their powerful automatic feature representation learning capabilities, data-driven deep learning methods for trajectory representation tend to have poorer interpretability. Apart from the aforementioned methods, there has also been some research on using natural language and graphic images to represent trajectories [39].

**Figure 1:**
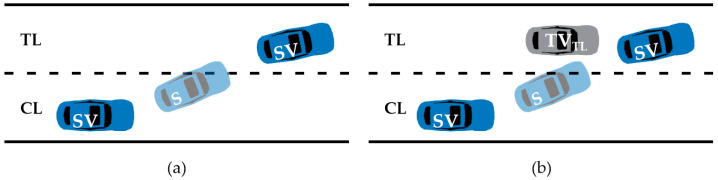
Comparison of two trajectory representation methods. (**a**) A trajectory representation method that only considers the target vehicle. (**b**) A trajectory representation method that considers the surrounding vehicles.

Despite some research having been conducted on vehicle trajectory representation, challenges persist [40]. As shown in Figure 1a, the existing trajectory representations described above mainly focus on describing the motion information of individual subject vehicles (SV), such as speed and position. However, during the actual lane-changing process, if the subject vehicle (SV) is too close to the vehicles behind it in the target lane (TV_TL_), leading to malicious lane-changing behavior, as illustrated in Figure 1b, the existing trajectory representation methods cannot accurately describe it. Therefore, existing trajectory representations suffer from the issue of being centered around a single subject vehicle, resulting in generated trajectories lacking interaction information between vehicles or targets.

The direct purpose of acquiring vehicle trajectories is to extract feature metrics and apply them to detection models. Many studies have focused on using physical quantities and their variants such as speed and displacement as the features of vehicle trajectories for subject vehicles. Regarding lane-changing behavior, studies in the past have concentrated on key factors like lane-changing frequency, speed, acceleration, and headway distance [41]. Yao et al. further refined these physical quantities by using their statistical measures, such as mean, extremum, and percentiles, rendering the trajectory features more nuanced [42]. In recent years, interactions between vehicles have also been considered in the selection of feature metrics, such as gaps and relative speeds with surrounding vehicles [43,44]. This has been applied to the study of lane-changing behavior, primarily throughout the entire lane-changing process. Thus, there is a lack of targeted research on feature extraction for different stages of lane-changing.

In terms of behavior detection, various machine learning algorithms such as neural network models [45,46], Support Vector Machine (SVM) [47,48,49], Dynamic Bayesian Networks [50,51], and Light Gradient Boosting Machine (LGBM) have been widely employed [52]. Among these, neural network models, due to their complexity, can lead to increased training times and potential entrapment in local or global optima [53]. Support Vector Machine (SVM), as a powerful tool for data classification, has extensive applications in pattern recognition research [54], but it is computationally complex and performs poorly when the sample sizes of various classes in the dataset are imbalanced. Dynamic Bayesian Networks are similarly computationally complex and handling high-dimensional data can be challenging. The LGBM algorithm, introduced in 2017, exhibits advantages over traditional algorithms, including low memory usage, higher accuracy, and the ability to handle high-dimensional and large-scale data [55].

In summary, despite the extensive research in trajectory representation and behavior detection methods, the existing studies exhibit the following gaps:

(1) The majority of existing trajectory representation studies are based on the subject vehicle, with limited consideration for surrounding vehicles. A trajectory representation method that integrates information from surrounding vehicles has not been proposed. Moreover, the selected trajectory feature indicators are mostly applied to the entire lane-changing process, lacking targeted proposals or selections for different stages of lane-changing.

(2) Compared to traditional machine learning models, the Light Gradient Boosting Machine (Light GBM) algorithm is better suited for the classification and detection of large-scale, high-dimensional data.

To address these gaps, we first propose a trajectory representation method that integrates information from surrounding vehicles. Subsequently, we define feature indicators that characterize different stages of lane-changing using this method. We summarize and categorize three typical instances of abnormal lane-changing behavior, followed by the application of the Light Gradient Boosting Machine (Light GBM) algorithm for the detection of abnormal lane-changing behavior.

The organization of this paper is as follows: The Section 2 outlines the data sources, trajectory representation method, feature extraction approach, and the establishment of the model for detecting abnormal lane-changing behavior. The Section 3 presents the experimental results and discussions. Finally, the Section 4 provides the conclusion and outlines future work.

## 2. Methodology

### 2.1. Research Framework

This study leverages roadside video data to propose a trajectory representation method that integrates information from surrounding vehicles. It combines machine learning techniques for detecting abnormal lane-changing behavior. This process encompasses three key tasks:

(1) Trajectory representation and feature extraction—achieving a trajectory representation that integrates information from surrounding vehicles. This involves considering the mutual interactions between vehicles and extracting features at different stages of lane-changing. Subsequently, lane-changing events are further extracted.

(2) Labeling of abnormal lane-changing behavior: three typical instances of abnormal lane-changing behavior are defined. A dataset of abnormal lane-changing behavior is established for training the model.

(3) Model establishment for abnormal lane-changing detection: the framework is designed to detect abnormal lane-changing behavior, leveraging the dataset established in the previous step.

**Figure 2:**
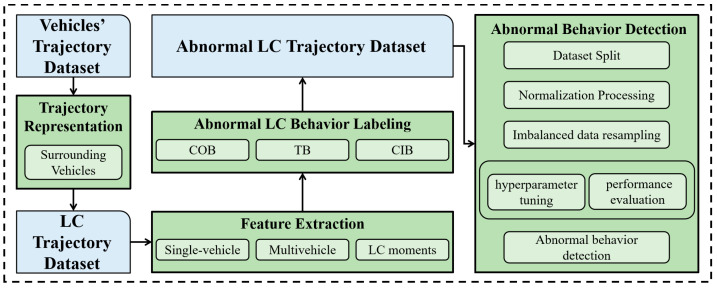
Research framework.

The research framework is illustrated in Figure 2.

### 2.2. Data Source

This study selected the Luoshi Road elevated expressway in Wuhan City as the observation site. Six observation points were established by six experimenters at elevated writing platforms on the roadside, equipped with fixed motion cameras. The observation scope covered the section from Bayi Road to Mafangshan Tunnel, with a total effective road length of 1.41 km, including various types of sections such as weaving zones, merging, and diverging areas. After careful screening and inspection of the videos captured at the six points, valid videos from 14:30 to 18:00 were retained.

**Figure 3:**
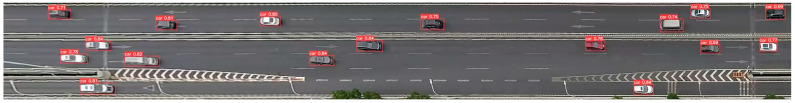
Vehicle detection at Point 3.

At Point 3, the length of the observed road segment was 205 m, with a dual-lane upstream section and a downstream section with four lanes, including merging and diverging areas. Due to the higher frequency of lane-changing events in the downstream section at this point, as well as the diversity of lane-changing types, this study chose the data from the downstream section at Point 3 to construct the vehicle trajectory dataset for further research. Figure 3 illustrates the vehicle detection process after perspective transformation of the captured images at Point 3. This study employed a Cartesian coordinate system to describe the trajectories, with the Y-coordinate defined as parallel to the direction of the lanes and the X-coordinate defined as perpendicular to the lane direction. The origin of the coordinates was set at the lower-left corner of the road section at Point 1, with the coordinate unit in terms of pixel values from the images. After applying perspective transformation to the dataset, performing vehicle object detection, and associating data, a total of 11,168 valid vehicle trajectories were extracted, providing motion information including vehicle position, speed, acceleration, etc. (at intervals of 0.1 s).

### 2.3. Trajectory Representation and Feature Extraction

**Definition 1** **(Trajectory Point).** *The trajectory of a vehicle at time i is represented as Ti = (S_i_,t_i_), where s_i_ is a sequence of pairs representing the precise spatial locations of the vehicle’s trajectory points and t_i_ is the timestamp indicating the time index of the trajectory point.*

**Definition 2** **(Trajectory).** *In geographical space, a time series of position sampling points with a certain time interval that can approximate the actual movement process of a mobile object is referred to as a trajectory. It can be expressed as a finite time series. In this paper, the trajectory represents the vehicle’s driving process on the road:*

(1) $$T=(T_{1},T_{2},T_{3},\ldots ,T_{i},\ldots ,T_{n}),i\in \left[1,n\right]$$

**Definition 3** **(Lane ID).** *The detection range of the roadside perception unit is fixed, and the lane position is fixed. Therefore, the current lane ID of the target vehicle can be identified based on the vehicle’s position (e.g., centroid position). The Lane ID defined in this paper is the lane ID where the target vehicle is located.*

**Figure 4:**
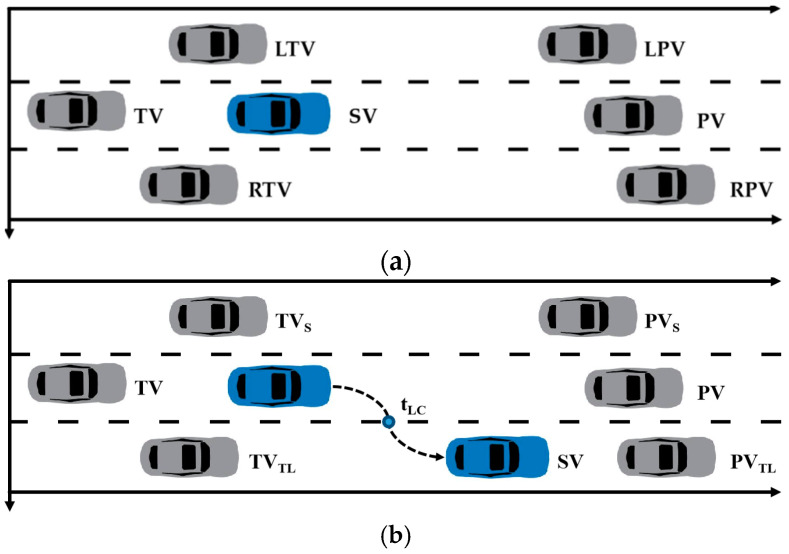
Illustration of surrounding vehicles. (**a**) trajectory representation. (**b**) lane change feature extraction.

**Definition 4** **(Surrounding Vehicles).** *By utilizing the roadside perception unit, the positions of all vehicles within the perception range can be extracted. Based on this, the trajectory representation method proposed in this study integrates information from surrounding vehicles. The definition of surrounding vehicles is shown in Table 1. This method is universally applicable, suitable for both the following process and lane-changing process of vehicles. Furthermore, this study focuses on the research of abnormal behaviors during the vehicle lane-changing process and provides specific definitions and descriptions for surrounding vehicles in the lane-changing process (refer to Table 1). Corresponding illustrations are shown in Figure 4, where Figure 4a is applied to trajectory representation and Figure 4b is applied to lane change feature extraction.*

**Definition 5** **(Trajectory Representation).** *In this paper, considering the integration of information regarding the positions of surrounding vehicles and the lane information, a novel method for representing vehicle trajectories is proposed. Specifically, it employs (S_SV_, S_LPV_, S_LTV_, S_PV_, S_TV_, S_RPV_, S_RTV_) to represent the positional information of the target vehicle and surrounding vehicles, where t_i_ represents the timestamp and r indicates the lane ID. The specific form is as follows:*

(2) $$\begin{array}{l} T_{i}^{\prime }=\left(s_{SV},s_{LLV},s_{LTV},s_{LV},s_{TV},s_{RLV},s_{RTV},t_{i},r\right)\\ T^{\prime }=(T_{1}^{\prime },T_{2}^{\prime },T_{3}^{\prime },\ldots T_{i}^{\prime },\ldots T_{n}^{\prime }),i\in \left[1,n\right] \end{array}$$

**Definition 6** **(Lane-Change Feature Extraction).** *The proposed trajectory representation method allows for the extraction of characteristic variables that represent the interactions between vehicles during lane-changing processes. These features can be directly computed from the trajectory representation results. For instance, variables like v and a can be obtained from the instantaneous positions of both the target vehicle and surrounding vehicles. The occurrence time of a lane change can be determined by checking if the lane ID has changed.*

The longitudinal velocities of vehicles SV, PV, PV_TL_, TV_TL_, PV_S_, and TV_S_ are defined as *v*, *v*_1_, *v*_2_, *v*_3_, *v*_4_, and *v*_5_, respectively. The relative speed between vehicles is defined as follows:

(3) $$\left\{\begin{array}{l} v_{r1}=v_{1}-v\\ v_{r2}=v_{2}-v\\ v_{r3}=v-v_{3}\\ v_{r4}=v_{4}-v\\ v_{r5}=v-v_{5} \end{array}\right.,$$

Lane-changing behavior is a continuous process, typically lasting several seconds, encompassing the driver’s decision-making, preparation, and the execution and completion of the lane change. Information such as the positions and speeds of surrounding vehicles before a lane change affects the driver’s decision and preparation. Additionally, the states of preceding and following vehicles after a lane change influence the progression and duration of the driver’s lane-changing action. Furthermore, the driver’s driving habits and style also impact the behavioral performance of the entire lane-changing process. Therefore, this study primarily considers four factors in selecting feature indicators:

(1) Multi-vehicle variables, obtained through trajectory representation calculations that integrate information from surrounding vehicles.

(2) Single-vehicle variables, taking into account the influence of driver habits and style on the entire lane-changing process.

(3) Before-lane-change features, considering the impact of surrounding vehicles on the driver’s lane change preparation and decision-making before a lane change.

(4) After-lane-change features, considering the partial impact of surrounding vehicles on the driver’s completion of the lane change and the subsequent lane-keeping process.

For each LC event, a total of 156 candidate feature indicators were selected. Table 2 provides detailed descriptions of these candidate features.

### 2.4. Abnormal LC Behavior Labeling

In this study, lane-changing trajectories were extracted from trajectory data obtained through video analysis. Both normal and abnormal lane-changing behaviors were annotated based on original video footage. The specific steps are outlined below:

Step 1: Constructing trajectories by integrating surrounding vehicle information. Surrounding vehicle data, including the position and lane ID of surrounding vehicles as defined in Table 1, were extracted. These were then represented in the form of trajectories as proposed in Section 2.3. If a vehicle was not present in a specific position around the SV vehicle, the relative positional information was set as infinity.

Step 2: Labeling of lane-changing events. For each trajectory, lane changes were determined based on lane IDs. If a change in lane ID was identified, the trajectory was marked as a lane-changing trajectory. Furthermore, the target lane of the vehicle was determined based on the change in lane ID.

Step 3: Extraction of lane-changing segments. Previous studies have indicated that the duration of lane-changing events does not exceed 5 s, with lane-changing processes occurring between 2 to 6 s constituting nearly 95% of cases. Additionally, the preparation time for lane changes takes approximately 2 s [56]. As this study primarily focuses on abnormal behaviors occurring before and after lane changes rather than the intention to change lanes, a reference point *t_LC_* was established based on the time of lane ID change. A fixed time duration of 6 s was employed to extract different lengths of time windows both before and after *t_LC_* (3 s before lane change +3 s after, 2 s before +4 s after, and 4 s before +2 s after). This facilitated the acquisition of three sets of lane-changing segments, allowing for the assessment of which window setting yielded the most effective detection of abnormal lane-changing behavior.

Step 4: Removal of Incomplete Segments. For each lane-changing trajectory, if the length before or after timestamp *t_LC_* does not meet the requirements of the specified time window, that lane-changing segment is deleted. This ensures uniformity in the length of extracted lane-changing segments (6 s) within each set, with consistent lengths before and after the lane change.

Step 5: Computation of Feature Parameters. Using the candidate feature parameters provided in Table 2, feature parameters are computed for each lane-changing segment.

**Figure 5:**
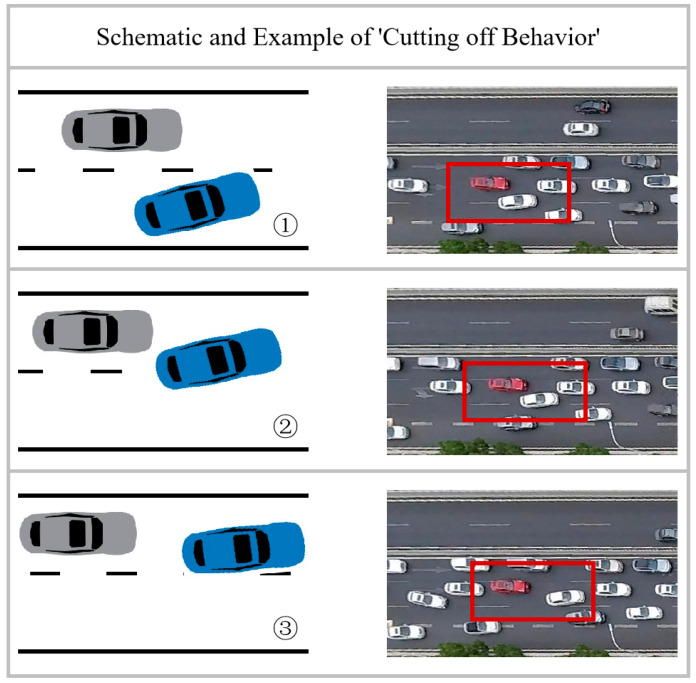
Schematic and example of “Cutting off behavior”.

**Figure 6:**
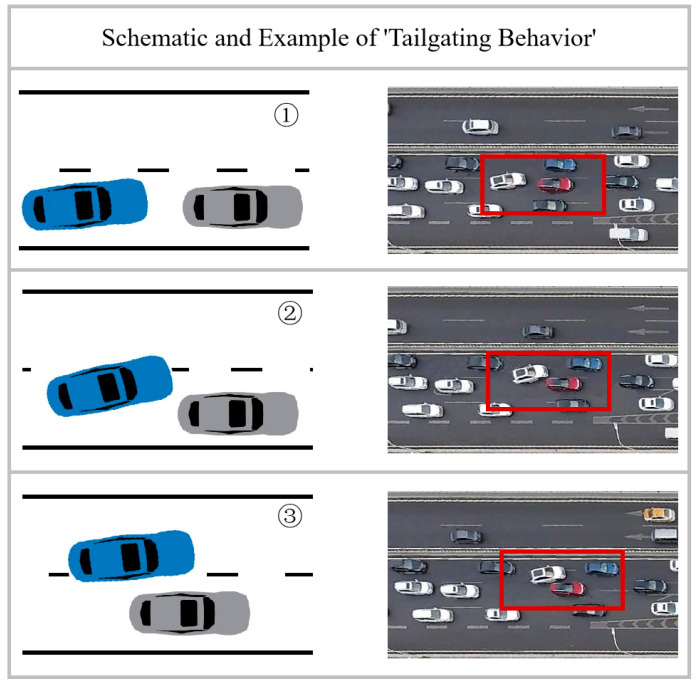
Schematic and Example of “Tailgating behavior”.

**Figure 7:**
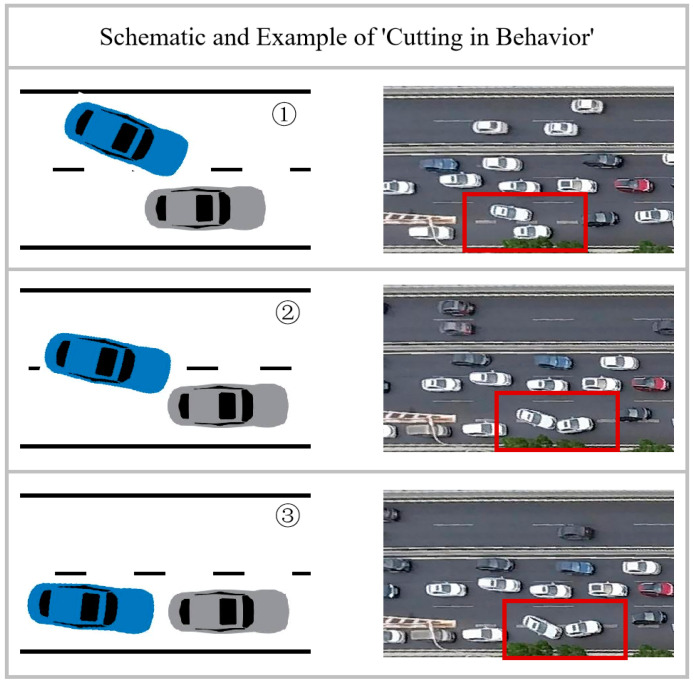
Schematic and Example of “Cutting in behavior”.

Step 6: Labeling Abnormal Trajectories. In this study, based on the following criteria, three types of lane-changing behaviors are classified as abnormal lane changes:

(1) “Cutting off behavior” (COB): In this study, the term “Cutting off behavior” is defined as a situation where a vehicle changes lanes with insufficient distance from the trailing vehicle in the target lane, leading to interference with the trailing vehicle’s movement. Figure 5 illustrates this concept, with a schematic on the left and an actual case on the right. The white vehicle within the red box changes lanes in close proximity to the red car, resulting in an emergency brake by the red car and a brief congestion behind it.

(2) “Tailgating behavior” (TB): In this study, “Tailgating behavior” is defined as a scenario where a vehicle in the lane-changing process maintains a close distance to the leading vehicle in the current lane. Figure 6 demonstrates this, with the white vehicle within the red box initiating and completing a lane change in close proximity to the red car in the adjacent lane. This behavior reduces the driver’s reaction time in emergency situations and increases the risk of collisions.

(3) “Cutting in behavior” (CIB): In this study, “Cutting in behavior” is defined as a situation where a vehicle changes lanes with insufficient distance from the leading vehicle in the target lane. Figure 7 provides a visual representation, showing a white vehicle within the red box forcefully completing a lane change without allowing adequate safety distance for the leading and trailing vehicles in the target lane, resulting in driving risks.

The annotation process was completed using a combination of manual labeling and trajectory data screening. The purpose of data screening was to extract potential segments of abnormal lane-changing behavior, thereby reducing the labor intensity of manual verification. The screening criteria were as follows:

(1) For COB behavior, it was required that there be a preceding vehicle in the target lane behind the subject vehicle. For TB behavior, the presence of a preceding vehicle in front was required. For CIB behavior, the presence of a vehicle in the front of the target lane was necessary.

(2) Surrounding vehicles were defined as those with a longitudinal distance less than 75 m from the subject vehicle [57].

(3) Both the subject vehicle and surrounding vehicles were required to have speeds greater than 1 m/s, ensuring that both vehicles were always in motion.

To enhance the accuracy of the labeling results, each video was processed by two individuals simultaneously. In cases of discordant opinions in the labeling results, the workers engaged in discussions to reach a consensus. Furthermore, the manual verification process adhered to the principle of accepting a high abnormality rate (above 10%) to ensure that valid events were not overlooked.

Due to the use of three different window settings, the number of segments varied under each setting. For instance, if a lane change occurred in the initial phase of a trajectory, resulting in a window before the lane change of less than 3 s, it would not be included in the “3 s before LC + 3 s after LC” segment set. Accordingly, the number of extracted lane-changing events for the three different window settings were 9313, 9102, and 9146. These events were then subjected to feature parameter computation and abnormal lane-changing behavior detection.

Each lane-changing segment could exhibit characteristics of more than one type of abnormal lane-changing behavior. As a result, the total count of normal and abnormal events exceeded the total count of lane-changing segments. Based on the different window settings, three distinct sets of segments were ultimately formed, named as Window Settings 1, Window Settings 2, and Window Settings 3. Table 3 summarizes the statistical results for these three datasets.

### 2.5. Abnormal Behavior Detection

In this study, the Light Gradient Boosting Machine (LGBM) algorithm was employed for detecting abnormal lane-changing behavior. Proposed by Microsoft in 2017, LGBM is a gradient boosting algorithm characterized by low memory usage, high efficiency, and strong interpretability [58]. The fundamental concept of the LGBM algorithm involves discretizing continuous floating-point feature values into k integers. It then constructs a histogram of width k, accumulating statistics based on the discretized values in the histogram. Ultimately, it identifies the optimal splitting point. Additionally, LGBM employs a leaf-wise growth strategy with depth limitations, replacing the traditional level-wise decision tree growth strategy. This enhancement improves accuracy while mitigating the risk of overfitting. The specific steps are outlined as follows:

Step 1: Train–Test Split. The samples were partitioned in a 7:3 ratio for training and testing, respectively. Seventy percent of the dataset was utilized for model training, followed by model evaluation on the remaining 30%.

Step 2: Normalization Process. Given that the units of the trajectory feature parameters used in this study varied, a min–max standardization method was employed to normalize the sample data, thereby eliminating disparities among features:

(4) $$x^{\prime }=(x-x_{\min})/(x_{\max}-x_{\min})$$

where *x*′ represents the normalized value, *x* denotes the original sample value, *x*_min_ stands for the minimum sample value, and *x*_max_ signifies the maximum sample value.

Step 3: Imbalanced Data Resampling. The imbalanced distribution of samples across different categories of lane-changing behaviors can impact the performance of the detection model. To mitigate this, the Synthetic Minority Oversampling Technique (SMOTE) method was applied to oversample the small sample data related to abnormal behaviors, thus rectifying the issue of sample imbalance.

Step 4: Hyperparameter Tuning. The Differential Evolution Algorithm (DE) was employed for fine-tuning the model to obtain the optimal hyperparameters for the LGBM model. These optimal hyperparameters were subsequently utilized for retraining the training dataset. Table 4 shows the results of LGBM model structure optimization based on the differential evolution algorithm.

Step 5: Abnormal Behavior Detection. The retrained LGBM model was applied to the test set to detect abnormal lane-changing behavior. Based on the test set’s results, a confusion matrix was obtained.

Step 6: Model Performance Evaluation. The model’s performance was evaluated using four metrics: precision, recall, accuracy, and F1 score.

## 3. Results and Discussion

### 3.1. Results of Abnormal LC Recognition

#### 3.1.1. Comparison of Model Performance with Three Different Window Settings

Modeling was conducted using the three sets of segments outlined in Section 2.4. These sets were partitioned into training and testing data in a 7:3 ratio. This resulted in respective test set sizes of 2794, 2731, and 2744. The LGBM model was implemented using the scikit-learn and LGBM libraries in Python 3.11.

Since each lane-changing segment could potentially involve multiple types of abnormal lane-changing behavior, the model established in this study conducted separate detection for the three types of abnormal lane-changing behavior. Specifically, it produced “yes” or “no” outcomes for each type.

**Figure 8:**
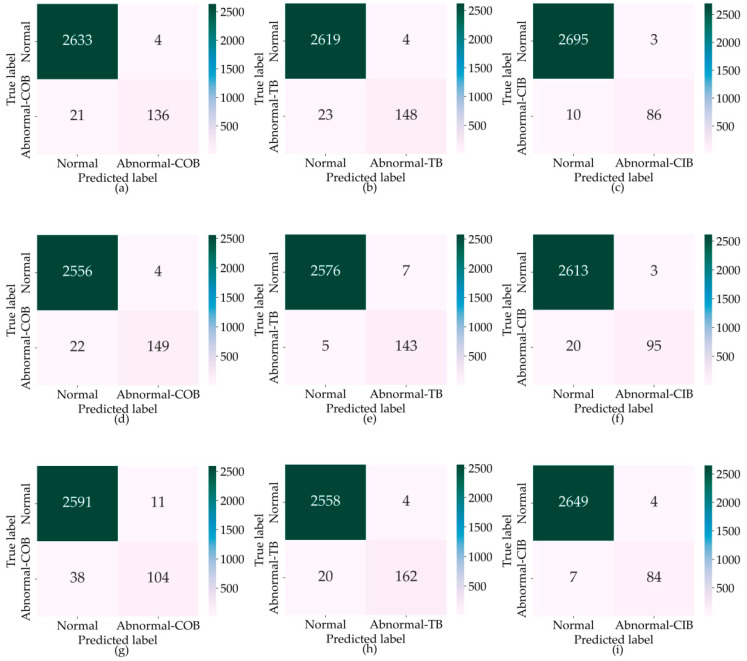
Confusion matrix of abnormal LC behavior detection using LGBM model. (**a**–**c**) represents the results of detecting COB, TB, and CIB using Window Settings 1, (**d**–**f**) represents the results of Window Settings 2, (**g**–**i**) represents the results of Window Settings 3.

Figure 8 illustrates the confusion matrices for the detection of abnormal lane-changing behaviors. Figure 8a–c represents the results of detecting COB, TB, and CIB using Window Settings 1 for training and testing. Figure 8d–i depicts the results for abnormal lane-changing behavior detection using Window Settings 2 and Window Settings 3, respectively. Larger values in the top-left corner of each confusion matrix indicate better performance in detecting “normal” behavior. Conversely, larger values in the bottom-right corner indicate superior performance in detecting “abnormal” behavior. Specifically, the top-left corner of each confusion matrix represents “True positive”, indicating the number of samples with “normal” behavior detected correctly. As shown in Figure 8, the True positive values for Window Settings 1 are all above 2600, with the highest for CIB behavior at 2695 and the lowest for TB behavior at 2619. The True positive values for Window Settings 2 and Window Settings 3 are lower than those under Window Settings 1 for the same behavior type, reflecting the superior detection performance of Window Settings 1 in normal behavior. The True positive values for COB and TB behaviors are both below 2600, and for CIB behaviors, they are 2613 and 2649, respectively. The bottom-right corner of the confusion matrix represents “True negative”, indicating the number of samples with “abnormal” behavior detected correctly. For COB behavior, Window Settings 2 has the highest True negative values, followed by Window Settings 1. For TB behavior, Window Settings 3 has the highest True negative values, followed by Window Settings 1. For CIB behavior, Window Settings 2 has the highest True negative values, followed by Window Settings 1. It can be seen that in terms of abnormal behavior detection, Window Settings 1 also shows good detection stability. In conclusion, since the True positive and True negative values under three window settings are relatively high, it indicates that the LGBM model used in this study performs well in the detection of abnormal lane-changing behaviors.

Quantitative analysis of the data in Table 5 reveals the following:

(1) Under all three window settings, the model’s accuracy is consistently above 0.9, and there is minimal variation in the detection results for each abnormal behavior across the three window settings, indicating good overall detection performance.

(2) In Window Settings 2, the precision for CIB detection is 0.826, lower than the 0.896 in Window Settings 1 and significantly lower than the 0.923 in Window Settings 1. In Window Settings 3, the precision for COB detection is 0.732, much lower than the 0.866 in Window Settings 1 and the 0.871 in Window Settings 2. This suggests that Window Settings 2 and Window Settings 3 exhibit shortcomings in the detection performance of CIB and COB behaviors, while Window Settings 1 demonstrates better detection stability.

(3) Under all three window settings, the model’s recall is consistently above 0.9, except for Window Settings 3, where the recall for COB behavior is lower than the other two settings. For the other two behavior types, there is minimal variation in the detection results across the three window settings.

(4) Window Settings 1 achieves F1 scores above 0.9 for all three abnormal behaviors, while Window Settings 2 shows F1 scores below 0.9 for CIB behavior, and Window Settings 3 exhibits F1 scores below 0.9 for COB behavior. Since F1 score best reflects the model’s comprehensive performance, Window Settings 1 demonstrates the best overall performance.

In summary, the models under all three window settings exhibit good detection levels, but Window Settings 1 show better detection stability and overall performance. Therefore, this study selects Window Settings 1, i.e., the “3 s before LC + 3 s after LC” window setting, as a representative choice for future investigations.

#### 3.1.2. Performance Comparison between LGBM Model and Three Other Typical Models

To further validate the performance of the proposed LGBM model, three widely recognized and commonly used methods were employed for comparison using the same segment dataset (Window Settings 1) and features. Table 6 presents the comparative results of the LGBM model with SVM, KNN, and XGBoost models in terms of two metrics. The following is shown in the table:

For the detection of “COB” behavior, the LGBM model exhibits a precision of 0.866 and an F1 score of 0.916, slightly lower than the XGBoost model’s 0.892 and 0.920, respectively, but higher than the SVM model and significantly higher than the KNN model.

For “TB” behavior detection, the LGBM model shows a precision of 0.865 and an F1 score of 0.916, slightly higher than the XGBoost model’s 0.858 and 0.899, and significantly higher than the SVM and KNN models.

In the case of “CIB” behavior detection, the LGBM model achieves a precision of 0.892 and an F1 score of 0.930, significantly higher than the XGBoost model’s 0.865 and 0.892, higher than the SVM model, and significantly higher than the KNN model.

Additionally, in terms of model training time, both the LGBM and XGBoost models demonstrate significantly shorter training times compared to the SVM and KNN models. Specifically, the XGBoost model has the shortest training time at 3.83 s, while the LGBM model’s training time is 4.07 s, 1.06 times that of the XGBoost model.

In summary, the following conclusions can be drawn: (a) The detection accuracy of the LGBM model is higher than that of the SVM model and significantly surpasses that of the KNN model; (b) The LGBM model exhibits slightly lower detection accuracy for “COB”, higher detection accuracy for “TB”, and significantly higher detection accuracy for “CIB” compared to the XGBoost model; (c) Both the LGBM and XGBoost models demonstrate relatively short training times, with the LGBM model’s training time slightly higher than the XGBoost model but significantly lower than the SVM and KNN models; (d) Considering the detection results of all four models, the LGBM model shows the optimal performance for detecting abnormal lane-changing behavior.

### 3.2. SHAP-Based Model Interpretation

**Figure 9:**
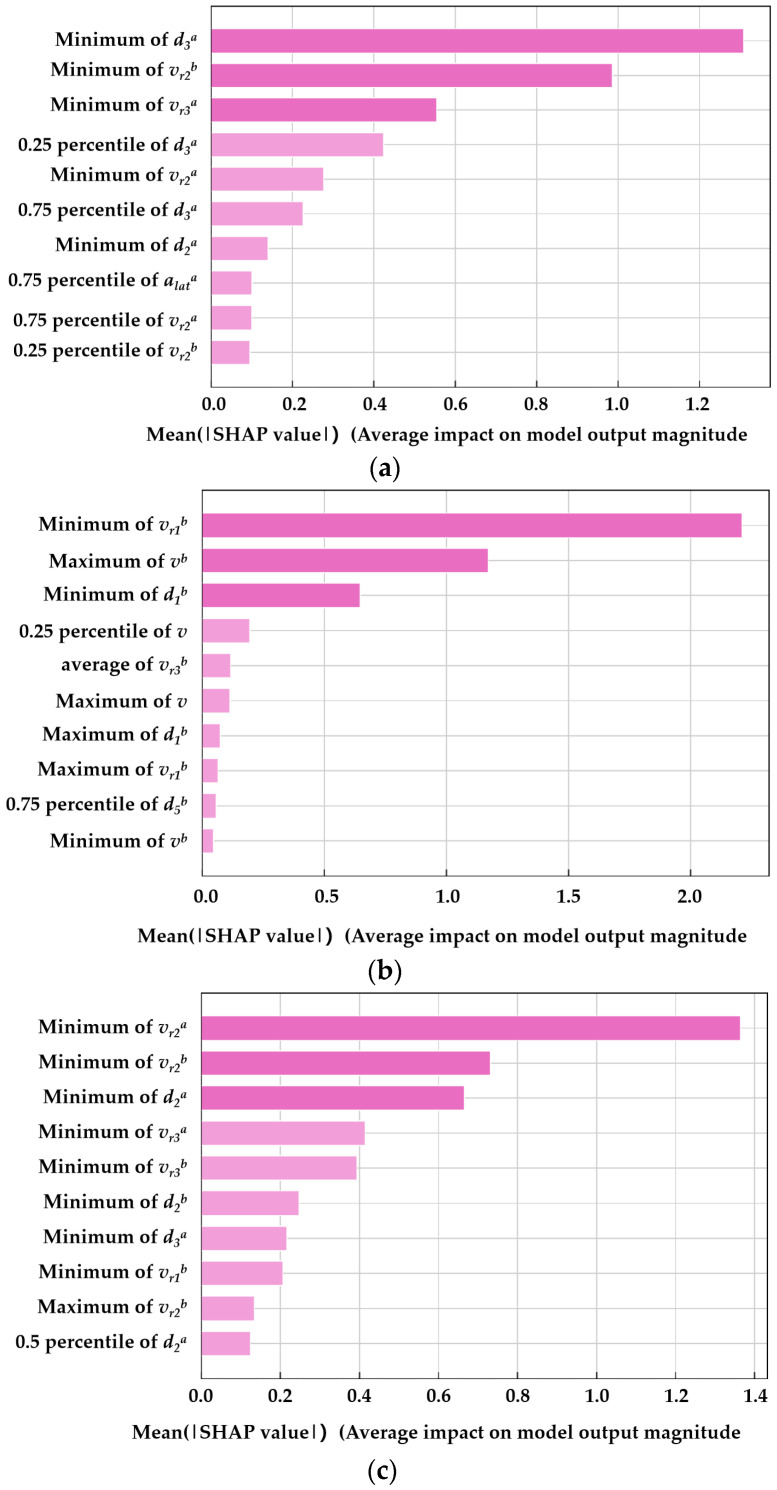
Overall importance of each feature variable. (**a**) shows the top ten features contributing to the detection of the three types of COB, (**b**) shows the top ten features contributing to the detection of the three types of TB, (**c**) shows the top ten features contributing to the detection of the three types of CIB.

The SHAP algorithm, proposed by Lundberg and Lee in 2017, can be used to understand the underlying mechanisms of machine learning models [59]. The core concept of SHAP values is to calculate the mean marginal contribution of a feature to the model’s prediction, thereby providing explanation and quantification of the model. Figure 9 displays the overall importance of each feature. Figure 9a–c show the top ten features contributing to the detection of the three types of COB, TB, and CIB abnormal behaviors, respectively. The horizontal axis represents the average SHAP value of the feature, while the vertical axis represents different features.

For COB behavior, the features with the highest contributions are the space headway between the subject vehicle and the trailing vehicle in the target lane after the lane change (*d_3_^a^*), the relative speed between the subject vehicle and the preceding vehicle in the target lane before the lane change (*v_r2_^b^*), and the relative speed between the subject vehicle and the trailing vehicle in the target lane after the lane change (*v_r3_^a^*). For TB behavior, the features with the highest contributions are the relative speed between the subject vehicle and the preceding vehicle in the same lane before the lane change (*v_r1_^b^*), the total longitudinal speed of the subject vehicle before the lane change (*v^b^*), and the space headway between the subject vehicle and the preceding vehicle in the same lane before the lane change (*d_1_^b^*). For CIB behavior, the features with the highest contributions are the relative speed between the subject vehicle and the preceding vehicle in the target lane after the lane change (*v_r2_^a^*), the relative speed between the subject vehicle and the preceding vehicle in the target lane before the lane change (*v_r2_^b^*), and the space headway between the subject vehicle and the preceding vehicle in the target lane after the lane change (*d_2_^a^*).

For the detection of each type of abnormal behavior, the features with the highest contributions follow these patterns: features with high contributions are mainly multi-vehicle variables, with the space headway and relative speed between vehicles being the most prominent; compared to entire lane change variables, before-lane-change variables and after-lane-change variables contribute significantly more; compared to other statistical values, the contributions of feature extremes (max and min values) are higher.

In summary, the following conclusions can be drawn:

(1) Features considering the relationships between vehicles, i.e., multi-vehicle variables, contribute far more to the detection of abnormal lane-changing behavior compared to single-vehicle variables. Multi-vehicle variables are calculated based on the proposed trajectory representation method that integrates surrounding vehicle information, underscoring the method’s importance in detecting abnormal lane-changing behavior;

(2) The distinct features before and after lane changes proposed in this study play a particularly significant role in the model;

(3) The contributions of space headway and relative speed extremes between vehicles are higher compared to other features.

**Figure 10:**
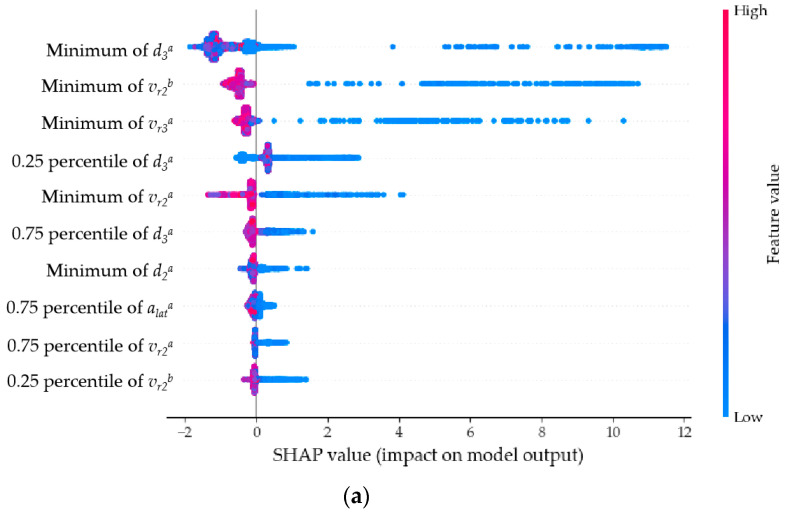

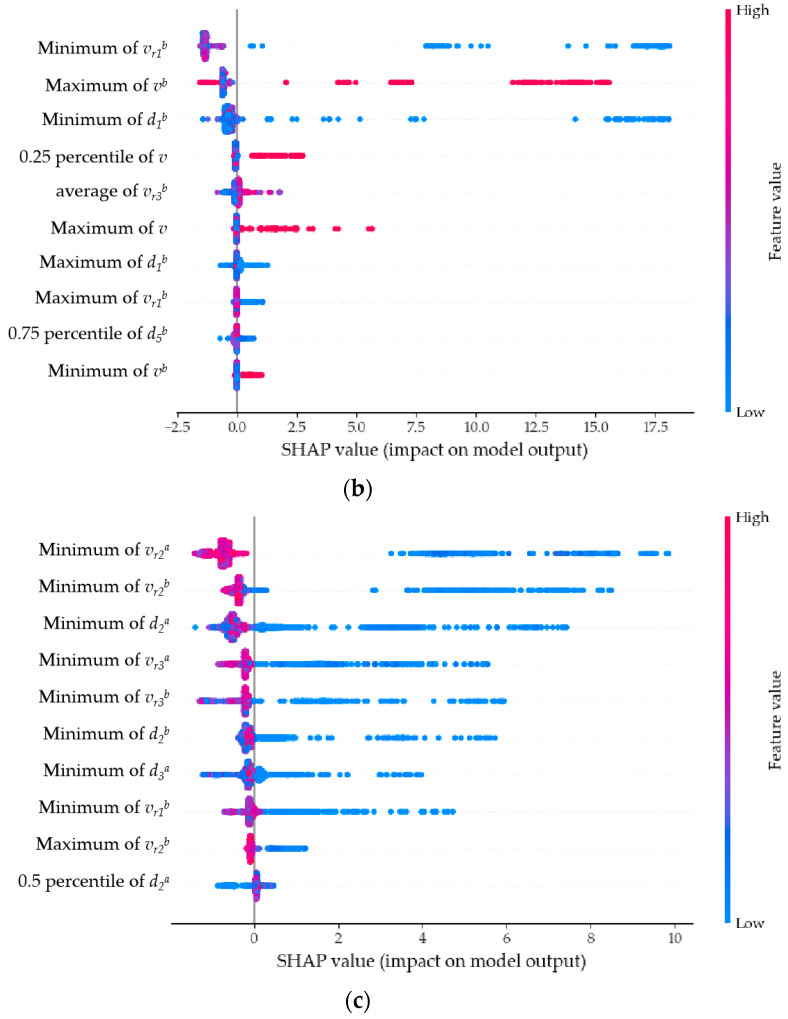
Distribution of SHAP values for each feature variable. (**a**) shows the results for COB, (**b**) shows the results for TB, (**c**) shows the results for CIB.

Figure 10 displays the specific impact of each sample feature on the detection model results within the SHAP interpretation framework. Figure 10a–c show the results for the three types of COB, TB, and CIB abnormal lane-changing behaviors, respectively. The vertical axis represents different features, while the horizontal axis represents the SHAP value of each sample feature. The depth of color corresponds to the color bar on the right, reflecting the magnitude of the sample feature value. For example, in Figure 10a, for the first three features, when the individual feature values are small, they exhibit large SHAP values, indicating a negative correlation. In Figure 10b, the second feature, maximum value of *v^b^*, shows the opposite pattern: when the individual feature value is large, the SHAP value is also large, indicating a positive correlation.

**Figure 11:**
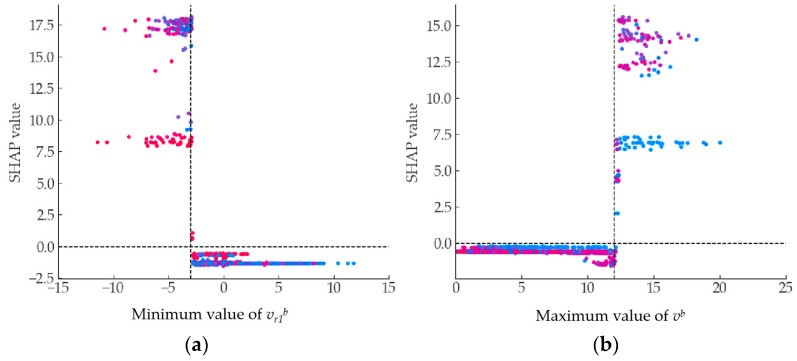
SHAP value mapping of the minimum value of *v_r1_^b^* and maximum value of *v^b^*. (**a**) shows the results for *v_r1_^b^*, (**b**) shows the results for *v^b^*.

Taking *v_r1_^b^* and *v^b^*, which contribute the most to TB, as an example, Figure 11 further illustrates the mapping relationship of individual feature variables with SHAP values. Figure 11a,b represent the minimum value of *v_r1_^b^* and maximum value of *v^b^* features, respectively. The horizontal axis represents the size of the feature variable’s value, while the vertical axis represents the SHAP value. The red dots in the figure indicate a significant contribution to the detection of abnormal behavior, while the blue dots indicate a significant contribution to the detection of normal behavior. When the minimum value of *v_r1_^b^* is greater than −3, the SHAP value is small, but when it is greater than −3, the contribution to both abnormal and normal behavior detection is significant. Conversely, for the maximum value of *v^b^*, the SHAP value is large when the feature value is greater than 12, and small when it is less than 12.

### 3.3. Discussion

In this paper, we innovatively defined three typical abnormal lane-changing behaviors: “Cutting off Behavior”, “Tailgating Behavior”, and “Cutting in Behavior”. While previous studies on abnormal driving behaviors have made significant progress, particularly in the use of driving simulators and naturalistic driving approaches, they have primarily focused on driver-operated behaviors, such as those induced by drivers’ psychology and style, for instance, “Talking on a cell phone while driving”, “Sounding the horn to express anger”, and the like [60]. Such behaviors are challenging for roadside cameras to capture, making them less applicable to road safety supervision. Guo et al. collect driving behavior data, extracting dangerous behaviors like “Sharp acceleration” and “Sharp left turn”, which were then applied to collision prediction [61]. However, the defined risky behaviors were limited to individual vehicles and did not consider interactions between vehicles. Wang et al. took into account the relationships between the changing vehicle and surrounding vehicles, extracting “Cutting in Behavior” for abnormal driving behavior [57]. Building upon this foundation, our study further defined three typical abnormal lane-changing behaviors.

Simultaneously, concerning the characterization of features related to abnormal lane-changing behavior, this paper also considers multi-vehicle factors, proposing “Single-vehicle variables” and “Multi-vehicle variables”. Additionally, the study accounts for different stages of lane-changing, introducing “Entire lane change variables”, “Before-lane change variables”, and “After-lane change variables”. Typically, parameters characterizing dangerous or abnormal driving behaviors focus on individual vehicle speed and acceleration, particularly acceleration [62]. Previous research has also suggested considering feature variables that capture the interactions between vehicles. For example, Xue et al. considered the gap and speed differences between vehicles [56], and Chen et al. expanded this to include acceleration differences between vehicles [63]. What sets this study apart is the use of “Multi-vehicle variables”, calculated based on the trajectory representation method proposed in this paper. Furthermore, the introduced features accounting for the before- and after-lane-change process, namely “Before-lane change variables” and “After-lane change variables”, make a significant contribution to the recognition of abnormal lane-changing behavior, playing a crucial role in the detection model presented in this paper.

In terms of detection models, machine learning models are crucial for conducting research on driving behaviors. Chen et al. employed the SVM method to identify hazardous driving behaviors such as Abrupt Double Lane Change (ALC), Retrograde Driving (RD), and Illegal U-Turn (IT), applying it to a video surveillance system [30]. Du et al. established a model using the KNN method and completed the lane change detection task using the NGSIM dataset [64]. Zhang et al. introduced a lane change prediction framework using the XGBoost model for lane-change behavior prediction [65]. In contrast, the LGBM model adopted in this study, proposed in recent years, has been demonstrated to exhibit high accuracy in the research on abnormal lane-changing behaviors presented in this paper.

The detection of abnormal driving is a significant contribution to the development of intelligent transportation. From the perspective of the driver, almost all vehicles collect speed data, and ADAS systems also issue warnings when the distance between two vehicles is too close. However, the results obtained in this study indicate that the relative speeds and distances between the front and rear vehicles in the same lane as well as the target lane can effectively characterize different forms of abnormal lane-changing behavior during lane changes. Therefore, the trajectory representation method and feature metrics proposed in this study provide a new approach for detecting abnormal driving behavior, which can be applied to autonomous driving.

Detecting abnormal driving behavior contributes significantly to the development of intelligent transportation. Therefore, from an applied perspective, this study can serve the domains of automated driving and traffic regulation in intelligent traffic environments. On one hand, from the driver’s perspective, nearly all vehicles collect speed data, and Advanced Driver Assistance Systems (ADAS) issue warnings when the following distance is too short. However, the results obtained in this study indicate that during lane-changing maneuvers, the relative speed and distance between the ego vehicle, leading vehicle in the current lane, and surrounding vehicles in the target lane can effectively characterize different forms of abnormal lane-changing behavior. Thus, the proposed trajectory representation method and associated features offer a new approach for abnormal driving behavior detection applicable to automated driving.

On the other hand, from a traffic regulation standpoint, this study focuses on lane-changing behavior, identifying three typical abnormal lane-changing behaviors through the detection model rather than merely distinguishing between normal and abnormal driving behavior. Furthermore, this research extends beyond significant violations or lapses, such as disregarding the speed limit [60], and starts from driving performance, identifying abnormal lane-changing behavior at the level of driving trajectories. This provides a new avenue for traffic authorities to detect and regulate dangerous driving behavior through roadside devices. Additionally, since this study is based on vehicle trajectory data, its applicability is not limited to roadside video data. Various methods for obtaining vehicle trajectories, including laser radar, millimeter-wave radar, unmanned aerial vehicle aerial photography, and sensor integration technologies, can all support the conduct and application of this research.

## 4. Conclusions

This paper proposes a trajectory representation method that integrates information about the positions of surrounding vehicles, diverging from the conventional trajectory representation based on an individual vehicle’s positional-time series; this research innovatively incorporates the relative positions between vehicles into the trajectory representation. Subsequently, this method is applied to roadside video data from expressways in Wuhan City. Utilizing the constructed driving trajectories, we introduce feature variables that take into account the interactions between vehicles and differentiate between before- and after-lane-change states. We then employ the LGBM model algorithm to detect abnormal lane-changing behavior and use the SHAP algorithm to interpret the influence of feature variables on the detection results. The results indicate that the LGBM model used in this paper outperforms other models in terms of detection performance, and the trajectory representation method and feature parameters proposed in this study make a significant contribution to the detection results.

In future work, we plan to conduct tests in different geographical locations using various sensor technologies to further enrich this study, thereby comprehensively improving the detection performance.

## Figures and Tables

**Table 1:** Definition and description of surrounding vehicles for trajectory representation and lane change feature extraction.

Trajectory Representation ^1^		Lane Change Feature Extraction ^2^		
Surrounding Vehicles	Description	Left LC	Right LC	Description
Subject Vehicle, SV	Subject vehicle in the scene	*SV*	*SV*	The subject vehicle, which changes lanes.
Preceding Vehicle, PV	Nearest forward vehicle in the same lane as the subject vehicle	*PV*	*PV*	The preceding vehicle of *SV* in the original lane.
Trailing Vehicle, TV	Nearest behind vehicle in the same lane as the subject vehicle	*TV*	*TV*	The trailing vehicle of *SV* in the original lane.
Left Preceding Vehicle, LPV	Nearest forward vehicle in the lane left of the subject vehicle	*PV_TL_*	*PV_S_*	The preceding vehicle of *SV* in the target lane when performing left lane change/the preceding vehicle of *SV* in the lane on the other side of the original lane when performing right lane change.
Left Trailing Vehicle, LTV	Nearest behind vehicle in the lane left of the subject vehicle	*TV_TL_*	*TV_S_*	The trailing vehicle of *SV* in the target lane when performing left lane change/the trailing vehicle of *SV* in the lane on the other side of the original lane when performing right lane change.
Right Preceding Vehicle, RPV	Nearest forward vehicle in the lane right of the subject vehicle	*PV_S_*	*PV_TL_*	The preceding vehicle of *SV* in the lane on the other side of the original lane when performing right lane change/the preceding vehicle of *SV* in the target lane when performing left lane change.
Right Trailing Vehicle, RTV	Nearest behind vehicle in the lane right of the subject vehicle	*TV_S_*	*TV_TL_*	The trailing vehicle of *SV* in the lane on the other side of the original lane when performing right lane change/the trailing vehicle of *SV* in the target lane when performing left lane change.

^1^ is the definition and description of surrounding vehicles in general situations, used for trajectory representation. ^2^ is a special definition for the lane-change feature extraction process.

**Table 2:** Explanation of the features during the lane change.

**Entire lane change variables**	
*v*	The longitudinal velocity of *SV* during the lane change (m/s)
*a*	The longitudinal acceleration of *SV* during the lane change (m/s^2^)
*v_lat_*	The lateral velocity of *SV* during the lane change (m/s)
*a_lat_*	The lateral acceleration of *SV* during the lane change (m/s^2^)
**Before lane change: Single-vehicle variables**	
*v^b^*	The longitudinal velocity of *SV* during the lane change (m/s)
*a^b^*	The longitudinal acceleration of *SV* during the lane change (m/s^2^)
*v_lat_^b^*	The lateral velocity of *SV* during the lane change (m/s)
*a_lat_^b^*	The lateral acceleration of *SV* during the lane change (m/s^2^)
**Before lane change: Multi-vehicle variables**	
*d_1_^b^*	The space headway between *PV* and *SV* (m)
*d_2_^b^*	The space headway between *PV_TL_* and *SV* (m)
*d_3_^b^*	The space headway between *TV_TL_* and *SV* (m)
*d_4_^b^*	The space headway between *PV_S_* and *SV* (m)
*d_5_^b^*	The space headway between *TV_S_* and *SV* (m)
*v_r1_^b^*	The relative speed of *PV* and *SV* (m/s)
*v_r2_^b^*	The relative speed of *PV_TL_* and *SV* (m/s)
*v_r3_^b^*	The relative speed of *TV_TL_* and *SV* (m/s)
*v_r4_^b^*	The relative speed of *PV_S_* and *SV* (m/s)
*v_r5_*	The relative speed of *TV_S_* and *SV* (m/s)
**After lane change: Single-vehicle variables**	
*v^a^*	The longitudinal velocity of *SV* during the lane change (m/s)
*a^a^*	The longitudinal acceleration of *SV* during the lane change (m/s^2^)
*v_lat_^a^*	The lateral velocity of *SV* during the lane change (m/s)
*a_lat_^a^*	The lateral acceleration of *SV* during the lane change (m/s^2^)
**After lane change: Multi-vehicle variables**	
*d_2_^a^*	The space headway between *PV_TL_* and *SV* (m)
*d_3_^a^*	The space headway between *TV_TL_* and *SV* (m)
*v_r2_^a^*	The relative speed of *PV_TL_* and *SV* (m/s)
*v_r3_^a^*	The relative speed of *TV_TL_* and *SV* (m/s)

1. For each characteristic indicator, calculate a set of statistical values consisting of mean, maximum, minimum, 0.25 quantiles, 0.5 quantiles (=median), and 0.75 quantiles. 2. Each lane change event is divided into two time periods: the before-lane-change period and the after-lane-change period.

**Table 3:** The statistics of LC segments.

		Window Settings 1	Window Settings 2	Window Settings 3
		3 s before LC + 3 s after LC	2 s before LC + 4 s after LC	4 s before LC + 2 s after LC
LC events		9313	9102	9146
Normal		8340	8085	8145
Abnormal	COB	521	569	507
	TB	574	513	578
	CIB	337	357	303

**Table 4:** Optimal Results of LGBM Based on DE algorithm.

Hyperparameters	Range	Optimization Results
Learning rate	(0.01,0.1)	0.059
Min data in leaf	(10,100)	67
Bagging fraction	(0.5,1)	0.965
Colsample bytree	(0.5,1)	0.983
Max depth	(5,20)	11
Lambda l1	(0,0.5)	0.0086
Lambda l2	(0,0.5)	0.3626
Min gain to split	(0,0.5)	0.2007
Scale pos weight	(1,10)	7

**Table 5:** Comparison of model performance with three different window settings.

	Window Settings 1			Window Settings 2			Window Settings 3		
	COB	TB	CIB	COB	TB	CIB	COB	TB	CIB
Precision	0.866	0.865	0.896	0.871	0.966	0.826	0.732	0.890	0.923
Accuracy	0.991	0.990	0.995	0.990	0.996	0.992	0.982	0.991	0.996
Recall	0.971	0.974	0.966	0.974	0.953	0.969	0.904	0.976	0.955
F1 score	0.916	0.916	0.930	0.920	0.960	0.892	0.809	0.931	0.939

**Table 6:** Performance comparison between LGBM model and three other typical models.

	COB		TB		CIB		Training Time/s
	Precision	F1 Score	Precision	F1 Score	Precision	F1 Score	
LGBM	0.866	0.916	0.865	0.916	0.896	0.930	4.07
SVM	0.833	0.864	0.824	0.829	0.808	0.843	12.73
KNN	0.706	0.793	0.748	0.806	0.659	0.733	9.48
XGBoost	0.892	0.920	0.858	0.899	0.865	0.892	3.83

## Data Availability

Data are contained within the article.
